# Differential Genetic Basis for Pre-Menopausal and Post-Menopausal Salt-Sensitive Hypertension

**DOI:** 10.1371/journal.pone.0043160

**Published:** 2012-08-17

**Authors:** Victoria L. M. Herrera, Khristine A. Pasion, Ann Marie Moran, Nelson Ruiz-Opazo

**Affiliations:** Section of Cardiovascular Medicine, Department of Medicine, Boston University School of Medicine, Boston, Massachusetts, United States of America; Karolinska Institutet, Sweden

## Abstract

Essential hypertension affects 75% of post-menopausal women in the United States causing greater cardiovascular complications compared with age-matched men and pre-menopausal women. Hormone replacement and current anti-hypertensive therapies do not correct this post-menopausal increased risk suggesting a distinct pathogenic framework. We investigated the hypothesis that distinct genetic determinants might underlie susceptibility to salt sensitive hypertension in pre-menopausal and post-menopausal states. To determine whether distinct genetic loci contribute to post-menopausal salt-sensitive hypertension, we performed a genome-wide scan for quantitative trait loci (QTLs) affecting blood pressure (BP) in 16-month old post-menopausal F2 (Dahl S×R)-intercross female rats characterized for blood pressure by radiotelemetry. Given identical environments and high salt challenge, post-menopausal BP levels were significantly higher than observed in pre-menopausal (post-menopausal versus pre-menopausal SBP, P<0.0001) and ovariectomized (post-menopausal versus ovariectomized SBP, P<0.001) F2-intercross female rats. We detected four significant to highly significant BP-QTLs (*BP-pm1* on chromosome 13, LOD 3.78; *BP-pm2* on chromosome 11, LOD 2.76; *BP-pm3* on chromosome 2, LOD 2.61; *BP-pm4* on chromosome 4, LOD 2.50) and two suggestive BP-QTLs (*BP-pm5* on chromosome 15, LOD 2.37; *BP-f1* on chromosome 5, LOD 1.65), four of which (*BP-pm2*, *BP-pm3*, *BP-pm4*, *BP-pm5*) were unique to this post-menopausal cohort. These data demonstrate distinct polygenic susceptibility underlying post-menopausal salt-sensitive hypertension providing a pathway towards the identification of mechanism-based therapy for post-menopausal hypertension and ensuing target-organ complications.

## Introduction

Cumulative observations indicate that sex-specific susceptibility exists in various traits [1]–[5] including essential hypertension [6], [7]. Moreover, essential hypertension affects a substantial proportion of post-menopausal women in United States approaching 75% of this population [8] with an increased prevalence of salt-sensitive hypertension [9]–[11]. Notably, hypertension-related cardiovascular complications are also greater in post-menopausal women compared with age-matched men despite equivalent anti-hypertensive intervention [12] and pre-menopausal women [13]. Several risk factors are thought to contribute to increased hypertension-risk in post-menopausal women, such as endothelial dysfunction, arterial stiffness, salt-sensitivity and obesity [14]. Multiple mechanisms have been postulated to underlie post-menopausal hypertension including estrogen/androgen ratios, increase in endothelin-1 and activation of the renin-angiotensin system [15]. However, no unifying pathogenic framework has been identified.

The increased prevalence of salt-sensitive hypertension in post-menopausal women [9]–[11] has been linked to the loss of endogenous estrogens based on observations showing that surgical menopause is associated with the development of salt-sensitive hypertension in previously healthy salt-resistant women [16]. However, recent reports of clinical estrogen/progestin replacement studies have not revealed similar benefits, but in fact have shown adverse cardiovascular events of hormone therapy in aging women [17]. These observations indicate that there is an inherent complexity of underlying mechanisms involved in increased salt-sensitive hypertension and cardiovascular disease after menopause, and suggest the hypothesis that distinct genetic determinants underlie post-menopausal salt-sensitive hypertension.

## Results

To investigate the genetic determinants that contribute to post-menopausal salt-sensitive hypertension we performed a genome scan for QTLs affecting blood pressure in an F2 (Dahl S×R)-intercross post-menopausal female rat population characterized for blood pressure by radiotelemetry. We implemented the following experimental design: Female F2 hybrids were maintained from weaning on a low salt (0.008% NaCl) diet until challenge with high salt (8% NaCl) at post-menopause confirmation at 14 months of age. BP-implant surgery was done at 13.5 months of age; after 12 days, baseline BP levels were obtained. We characterized 130 subjects for blood pressure. The cohort was genotyped with 100 informative markers for our (Dahl S×R) intercross with an average density of 23.9 Mbp.

**Figure 1:**
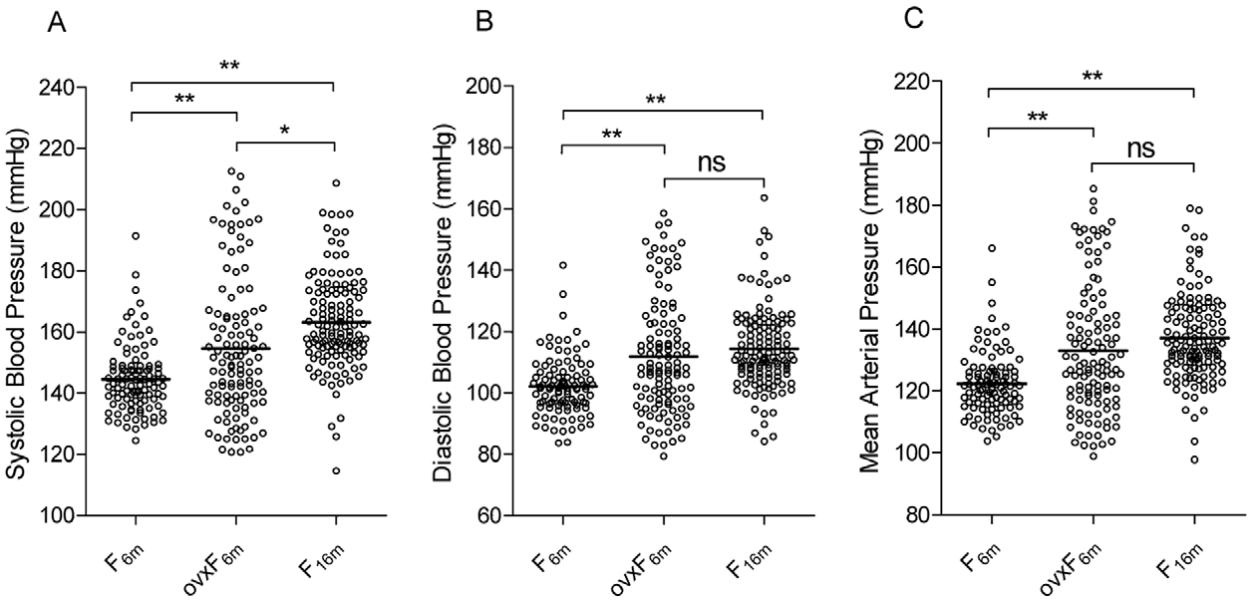
Distribution of BP in male, pre-menopausal and post-menopausal F2 (Dahl S×R)-intercross cohorts. Distribution of systolic (A), diastolic (B) and mean (C) blood pressures in pre-menopausal F2 (F_6m_, n = 102) female hybrids at 6 months of age (8% NaCl challenge begun at 3 months of age); ovariectomized female F2 (ovxF_6m_, n = 116) hybrids at 6 months of age (8% NaCl challenge begun at 3 months of age) and post-menopausal F2 (F_16m_, n = 130) female hybrids at 16 months of age (8% NaCl challenge begun at 14 months of age). Means are shown as horizontal lines. ^*^ *P*<0.001, ^**^ *P*<10^−4^ (one-way ANOVA followed by Tukey test for multiple comparisons).

We first analyzed the impact of menopause on salt-sensitive hypertension by comparing BP parameters between post-menopausal (Figure 1, F_16m_) and previously reported pre-menopausal (Figure 1, F_6m_) [7] and ovariectomized (Figure 1, ovxF_6m_) [18] F2 (Dahl S×R)-intercross rat cohorts. Concordant with observations in human studies [9]–[11], menopause increased systolic (SBP, *P*<10^−4^), diastolic (DBP *P*<10^−4^) and mean arterial pressures (MAP *P*<10^−4^) significantly compared with BP in pre-menopausal rats (Figure 1). This validates our F2 (Dahl S×R)-intercross as a biological post-menopausal F2-intercross cohort that recapitulates observations in humans. Compared to ovariectomized 6 m-old female F2-intercross rats, BP levels in 16 m-old post-menopausal rats is also higher (SBP, *P*<10^−3^, Figure 1).

**Figure 2:**
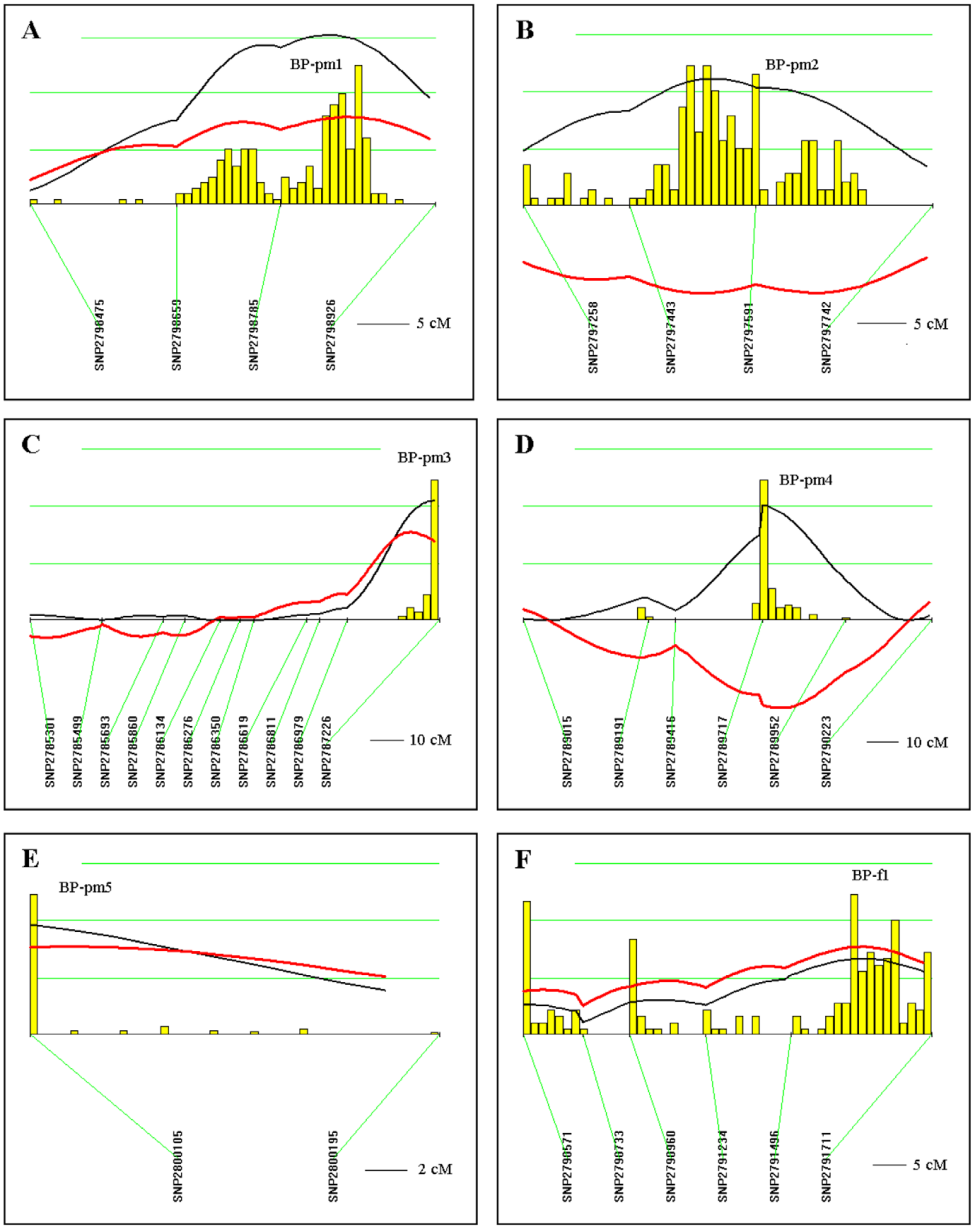
QTLs for blood pressure (BP) in post-menopausal (16 months old) F2 (Dahl S×R)-intercross female rats. Chromosomes with suggestive, significant and highly significant QTLs were analyzed by interval mapping with bootstrap resampling method to estimate a confidence interval (QTXb19 Map Manager): Panel A, chromosome 13 (*BP-pm1*); B, chromosome 11 (*BP-pm2*); C, chromosome 2 (*BP-pm3*); D, chromosome 4 (*BP-pm4*); E, chromosome 15 (*BP-pm5*) and F, chromosome 5 (*BP-f1*). Yellow histograms represent the bootstrap-based confidence intervals for the detected QTLs. For a histogram with single peak, widths define the confidence interval for the QTL. Histograms with more than one peak suggest that there may be multiple linked QTLs or that the QTL is not well defined (QTXb19 Map Manager). Orientation of chromosomes: left→right starting from lowest Mbp. Horizontal green lines [—] mark LOD values for significance of linkage, from top to bottom: highly significant LOD≥3.70; significant LOD≥2.48; suggestive LOD≥1.22; LOD [—]; regression coefficient [—].

We next performed a total genome scan for QTLs affecting blood pressure (BP) using 16 m-old post-menopausal F2 (Dahl S×R)-intercross female rats. Results were then compared to genome scan results obtained in 6 m-old pre-menopausal and 5 m-old male F2 (Dahl S×R)-intercross rats [7], as well as to observations on 6 m-old ovariectomized F2 (Dahl S×R)-female cohort [18]. We identified six BP QTLs with highly significant (n = 1), significant (n = 3) and suggestive linkage (n = 2) (LOD 1.65–3.78, Table 1). Notably, five out of six BP-QTLs (*BP-pm1* on chromosome 13, LOD 3.78; *BP-pm2* on chromosome 11, LOD 2.76; *BP-pm3* on chromosome 2, LOD 2.61; *BP-pm4* on chromosome 4, LOD 2.5 and *BP-pm5* on chromosome 15, LOD 2.37, Table 1, Figure 2) were not detected in pre-menopausal females (Table 2). One BP QTL (*BP-f1* on chromosome 5, LOD 1.65, Table 1, Figure 2) was previously detected in the pre-menopausal F2 (Dahl S×R) female cohort with significant linkage (Table 2). On the other hand, four out of six BP-QTLs (*BP-pm2*, *BP-pm3*, *BP-pm4* and *BP-pm5*, Table 1, Figure 2) were not detected in the ovariectomized F2 (Dahl S×R)-female cohort (Table 2). Two BP QTLs (*BP-pm1* and *BP-f1*, Table 1, Figure 2) were detected in the ovariectomized F2 (Dahl S×R) female cohort with suggestive linkage (Table 2). One post-menopausal BP QTL (*BP-pm2*, Table 1, Figure 2) was observed in the male F2 (Dahl S×R)-cohort study (*BP-m5*) detected with suggestive linkage (Table 2). Additional analysis for interactive effects on BP reveals no gene-gene interaction in the post-menopausal female F2-intercross cohort. In contrast, several interacting-loci were detected in pre-menopausal females affecting blood pressure that fulfilled the criteria for significant gene interaction [7].

**Table 1:** QTLs for blood pressure in post-menopausal F2 (Dahl S×R) intercross female rats.

*QTL*	*Model*	*Trait*	*Rat-location*	*LOD*	*%*	*Human-location*	*Human QTL*
*BP-pm1*	Recessive	SBP	Chr13:70–90 Mbp	3.78 (**HS**)	13 (↑)	Chr1:158–181 Mbp	BP5_H [19]
*BP-pm2*	Additive	SBP	Chr11:48–68 Mbp	2.76 (**S**)	9 (↓)	Chr3:104–124 Mbp	BP51_H [19]
*BP-pm3*	Recessive	DBP	Chr2:218–248 Mbp	2.61 (**S**)	9 (↑)	Chr1:81–103 Mbp	Detected [19]
*BP-pm4*	Additive	DBP	Chr4:103–121 Mbp	2.50 (**S**)	9 (↓)	Chr2:69–89 Mbp	BP10_H [19]
*BP-pm5*	Dominant	SBP	Chr15:21–26 Mbp	2.37 (**Sug**)	8 (↑)	Chr14:53–58 Mbp	
*BP-f1*	Additive	SBP	Chr5:135–156 Mbp	1.65 (**Sug**)	6 (↑)	Chr1:21–48 Mbp	EDN2 [25], [26]

Table legend: QTL, quantitative trait locus; SBP, systolic blood pressure; DBP, diastolic blood pressure; Chr, chromosome; %, the amount in % of total trait variance that would be explained by a QTL at these loci; Mbp, mega-base pair; LOD, logarithm of the odds score derived from the likelihood ratio statistic using a factor of 4.6; ↑, S-allele increases trait; ↓, S-allele decreases trait. Significance determined from 2000 permutations on data set: LOD 3.70 highly significant (HS); LOD 2.48 significant (S); LOD 1.22 suggestive (Sug). Human QTLs as per RGD.

**Table 2:** Post-menopausal BP QTLs detected in other F2 (Dahl S×R) intercrosses.

*16m*			*6m*			*6m*			*5m*		
*Post-menopausal*			*Ovariectomized * [*18*]			*Pre-menopausal * [*7*]			*Male * [*7*]		
QTL	SIG	Eff	QTL	SIG	Eff	QTL	SIG	Eff	QTL	SIG	Eff
*BP-pm1*	HSig	(↑)	*BP-fovx2*	Sugg	(↑)	-	-	-	-	-	-
*BP-pm2*	Sig	(↓)	*-*	-	-	-	-	-	*BP-m5*	Sugg	(↓)
*BP-pm3*	Sig	(↑)	*-*	-	-	-	-	-	-	-	-
*BP-pm4*	Sig	(↓)	*-*	-	-	-	-	-	-	-	-
*BP-pm5*	Sugg	(↑)	*-*	-	-	-	-	-	-	-	-
*BP-f1*	Sugg	(↑)	*BP-f1*	Sugg	(↑)	*BP-f1*	Sig	(↑)	-	-	-

Table legend: QTL, quantitative trait locus; *m*, months; SIG, significance; HSig, highly significant; Sig, significant; Sugg, suggestive; Eff, S-allele effect on trait; ↑, S-allele increases trait; ↓, S-allele decreases trait.

## Discussion

We found that the human syntenic regions corresponding to four of the six QTL regions detected in our post-menopausal cohort (Table 1) were also shown to influence blood pressure in a genome-wide linkage study of systolic and diastolic blood pressure performed in a Quebec Family Study [19], although sex-specific analysis was not reported. The regions span *BP-pm1* on rat chromosome 13, *BP-pm2* on rat chromosome 11, *BP-pm3* on rat chromosome 2 and *BP-pm4* on rat chromosome 4 (Table 1) suggesting that orthologous genes in these regions might underlie hypertension susceptibility in both post-menopausal Dahl rats and humans.

Given that loss or reduction in estrogen levels has been implicated as a key permissive factor in the increased incidence of hypertension and cardiovascular disease in post-menopausal women [15], [16], it was unexpected that clinical estrogen/progestin replacement studies would not lead to the expected benefits, but in fact showed adverse cardiovascular events of hormone therapy in aging women [17]. These polar clinical observations demonstrate the importance of systematic study in validated polygenic hypertension models of post-menopausal hypertension, such as in biological post-menopausal F2 (Dahl S×R)-intercross rats, in order to gain insight into the phenotype effects and genetic determinants of post-menopausal hypertension, while controlling for major confounders of hypertension, such as diet, genetic heterogeneity, environmental factors and developmental programming [20].

Consistent with increased salt-sensitive hypertension in post-menopausal women [10], comparative analysis of 6 m-old pre-menopausal, 6 m-old ovariectomized female and 16 m-old post-menopausal F2 (Dahl S×R)-intercross rats demonstrates that menopause increases salt-sensitive hypertension as a quantitative trait affecting systolic, diastolic and mean arterial pressures. Given the parallel studies using identical genetic F2-intercross design, the detection of multiple distinct BP QTLs among biological post-menopausal, pre-menopausal and surgical post-menopausal F2 (Dahl S×R)-intercross rats demonstrates that post-menopausal hypertension as modeled in the 16 m-old F2 (Dahl S×R) intercross rats involves genetic mechanisms not implicated in early-onset pre-menopausal and surgical post-menopausal salt-sensitive hypertension. We note that for some BP QTLs (*BP-pm2* and *BP-pm4*, Table 2) the S allele lowers blood pressure in the post-menopausal cohort in contrast to increasing blood pressure in all BP QTLs detected in pre-menopausal females [7] and most BP QTLs detected in males [7], except for *BP-m2* which is found in both post-menopausal and male populations having the same directional effect on BP, i.e., S allele decreasing BP [7]. These findings further indicate that differential genetic mechanisms underlie salt-sensitive hypertension in biological post-menopausal, pre-menopausal and surgical post-menopausal F2 genetic cohorts. Moreover, our results demonstrate an aging component to polygenic susceptibility to post-menopausal salt-sensitive hypertension since 6 months old ovariectomized females do not recapitulate the loci influencing blood pressure in 16 months old post-menopausal females.

Analysis of reported BP-QTLs in other Dahl S intercrosses using different normotensive strains revealed that *BP-pm1* and *BP-pm3* chromosomal regions overlapped with some BP-QTLs detected in other male and female intercrosses (Table 3). This suggests that genes underlying *BP-m1* and *BP-m3* might affect salt-sensitive hypertension susceptibility independently of sex, environment and age, although identification of corresponding gene variants will be necessary to verify this hypothesis.

**Table 3:** Post-menopausal significant and highly significant BP QTLs detected in other Dahl S intercrosses.

*QTL*	*Rat-location*	*QTL* a	*Rat-location* b	*Strain* c	*Sex*	*[Ref]*
*BP-pm1*	Chr13: 70–90 Mbp	Bp221	Chr13: 48–93 Mbp	Brown Norway	M	[27]
		Bp329	Chr13: 61–77 Mbp	Brown Norway	M+F	[28]
*BP-pm3*	Chr2: 218–248 Mbp	Bp175	Chr2: 223–256 Mbp	Brown Norway	F	[6]
		Bp202	Chr2: 146–232 Mbp	Brown Norway	M	[27]
		Bp239	Chr2: 236–258 Mbp	Milan Normotensive	M	[29]

Table legend: QTL, quantitative trait locus; Chr, chromosome; Mbp, mega-base pair; M, male intercross; F, female intercross; Ref, reference;

a , QTL name as per RGD (rat genome database);

b , rat-location as per RGD;

c , contrasting strain.

Altogether, detection of distinct post-menopausal BP QTLs, the failure of hormone replacement therapy to reverse clinical post-menopausal hypertension [17], and worse target organ complications despite equivalent anti-hypertensive interventions [12], suggests a putative de-repression paradigm. The loss of ovarian hormones and the ensuing post-menopausal microenvironment de-represses hypertension susceptibility genetic mechanisms, which then result in mechanistic set-point changes not reversed by hormone replacement therapy, similar to the non-reversibility of menopause by hormone replacement therapy [13]. On the other hand, the non-detection of several BP QTLs previously identified in a pre-menopausal F2 (Dahl S×R)-intercross cohort [7] suggest that these loci are ovarian hormone-dependent. These observations raise the hypothesis that presence or absence of ovarian hormones is critical in determining the specific genetic loci contributing to salt-sensitive hypertension and its target organ complications.

In conclusion, our study demonstrates the involvement of distinct genetic loci, and hence differential genetic mechanisms underlying susceptibility in pre-menopausal, post-menopausal and surgical post-menopausal salt-sensitive hypertension. While observations provide insight into the failure of hormone replacement therapy for post-menopausal salt-sensitive hypertension and its inadvertent worsening of cardiac events, more importantly these data provide compelling evidence to mandate the elucidation of genetic mechanisms in post-menopausal salt-sensitive hypertension as the *a priori* basis for much-needed prevention and intervention strategies.

## Materials and Methods

### Ethics statement

This study was performed in strict accordance with the recommendations in the Guide for the Care and Use of Laboratory Animals of the National Institutes of Health. The protocol was approved by the Committee on the Ethics of Animal Experiments of Boston University School of Medicine (Permit Number: AN-14966). All surgery was performed under sodium pentobarbital anesthesia, and every effort was made to minimize suffering.

### Genetic crosses

Inbred Dahl S/jrHsd and Dahl R/jrHsd rats were obtained from Harlan (Indianapolis, Indiana). Parental strains (Dahl R/jrHsd female×Dahl S/jrHsd male) were crossed to produce F1 progeny. The F2 subjects were derived from brother-to-sister mating of F1 hybrids to produce the F2 female (n = 130) segregating population.

### Ascertainment of post-menopausal status

In order to ascertain post-menopausal status of the female F2 hybrids to be characterized for blood pressure daily vaginal smears were performed on females to determine the stage of their estrous cycle (diestrous, proestrous or estrous) essentially as described [21], [22]. Female hybrids bred for phenotypic characterization were subjected to vaginal smears commencing at 12 months of age until 14 months of age. Cessation of cycling in the female hybrids was defined as continuous estrous for 4 weeks [21]. The post-menopausal F2 population showed that >99% of females stopped cycling by 14 months of age. Female subjects were maintained on a low salt (0.008% NaCl) diet until high salt (8% NaCl) challenge began at 14 months of age to avoid raises in blood pressure prior the high salt challenge.

### Blood pressure measurements

Blood pressure (BP) was measured essentially as described [7], [23] using intra-aortic abdominal radiotelemetric implants (DATASCIENCE) obtaining non-stressed blood pressure measurements taking the average over ten-seconds every 5 minutes for 24 hours [7], [23]. Systolic (SBP), diastolic (DBP) and mean arterial pressures (MAP) were obtained along with heart rate and activity. The protocol for the post-menopausal rats was as follows: implant surgery at 13 ½ months of age; only rats with no post-operative complications were used; after 12 days, baseline BP levels were collected. The high salt (8% NaCl) challenge was initiated at 14 months of age and maintained for four weeks. Females were maintained on a low salt (0.008% NaCl) diet until high salt (8% NaCl) challenge began at 14 months of age. BP values used for phenotype comparison were the averages obtained from the last 3 days of the fourth week of the salt loading from 24-hour recordings during no-entry (entry to BP room) or minimal entry days (Friday-Monday) ascertaining non-stress BP.

### Intercross linkage analysis

Phenotype distributions were analyzed for normality; data transformations were done when necessary and datasets that passed Kolmogorov-Smirnov normality testing (SigmaStat) were used for linkage analysis. QTL analysis was performed using SBP and DBP as quantitative traits. Linkage maps, marker regression and composite interval mapping were done with the Map Manager QTXb19 (MMQTXb19) program for windows [24] which generates a likelihood ratio statistic (LRS) as a measure of the significance of a possible QTL. Genetic distances were calculated using Kosambi mapping function (genetic distances are expressed in centiMorgan, cM). Critical significance values (LRS values) for interval mapping were determined by a permutation test (2000 permutations at all loci tested) on our post-menopausal female cohort using Kosambi mapping function and a dominant, recessive or additive regression model. Values for suggestive linkage LRS = 5.6 (LOD 1.22), for significant linkage LRS = 11.4 (LOD 2.48) and for highly significant linkage LRS = 17.0 (LOD 3.70). LRS 4.6 delineates LOD 1-support interval. Confidence interval for a QTL location was estimated by bootstrap resampling method wherein histogram single peak delineates the QTL and peak widths define confidence interval for the QTL. Histograms which show more than one peak warn that the position for the QTL is not well defined or that there may be multiple linked QTLs (QTX Map Manager). We also performed interaction analysis using the Map Manager QTXb19 program applying a two-stage test paradigm for determination of interaction in which the pair of loci must pass two tests in order to be reported as having a significant interaction effect. First, the significance of the total effect of the two loci must be <0.00001 and second, the pairs of loci must exhibit a *P* value<0.01 for the interaction effect.

### Genotyping

SNP genotyping was carried out on an Applied Biosystems 7900 Real-Time PCR System. SNPs (n = 97) and SSLP markers (n = 3) were selected from the RGD SNP database. SNP assays (TaqMan assays) were procured from Applied Biosystems and were validated in our laboratory.

### Statistical analyses

We performed one-way ANOVA followed by all pairwise multiple comparisons using Tukey test for blood pressures as indicated per experimental comparison.
